# Gamified eco-points and cost salience—when rewards “do” and “do not” align in anti-food-waste apps

**DOI:** 10.3389/fpsyg.2026.1686879

**Published:** 2026-04-02

**Authors:** Tingyun Luo, Juiche Tu, Lixia Liu, Chengxi Wang, Tao Yang, Xihui Jia, Ting Liu

**Affiliations:** 1 School of Design, Fujian University of Technology, Fuzhou, China; 2 Department of Creative Design, National Yunlin University of Science and Technology, Douliu, Yunlin, Taiwan; 3 College of Art, Beijing Union University, Beijing, China; 4 Graduate School of Design, National Yunlin University of Science and Technology, Douliu, Yunlin, Taiwan; 5 School of Fine Arts, Hubei Normal University, Huangshi, China

**Keywords:** food waste, gamification, platform trust, social media, sustainable food platforms

## Abstract

Anti-food-waste apps increasingly use gamified “eco-points” to nudge sustainable choices, yet when rewards align—or inadvertently fail—remains unclear, particularly under high price salience. This study examines how gamification, cost salience, and platform-trust cues shape consumer intentions toward sustainable food platforms. This study used an explanatory sequential mixed-methods design. First, three semi-structured expert interviews (interaction design, food co-op operations, and secondary education) were open-axial-selectively coded to surface design levers and pain points. Next, a cross-sectional survey of Taiwanese consumers (*N* = 256; 5-point Likert) assessed cognition, platform demand, reward perceptions, trust/traceability cues, assortment, and price acceptance. Reliability and construct adequacy were strong (Cronbach’s *α* = 0.949; KMO = 0.938; Bartlett’s *χ*^2^ = 5353.75, df = 435, *p* < 0.001). Exploratory factor analysis extracted four components (63.09% variance). Consumers endorse platform potential (*M* = 3.94), rate educational content (*M* = 4.10), and third-party product certificates/traceability highest (*M* = 4.27). Willingness to pay a price premium is modest (*M* = 3.32), indicating cost salience as a key barrier. Eco-points are viewed favorably (*M* = 3.92), but, in importance–performance map analysis (IPMA), rank below reliability information; transparency gaps (e.g., missing test reports or real-time verification) erode trust. The four-factor structure captures (1) sustainability cognition, (2) platform enablement, (3) reliability/traceability, and (4) cost/education salience. Occupation moderates platform demand, *F*(10,245) = 2.281, *p* = 0.014, with finance/insurance respondents showing higher demand than public-sector and retired groups. Rewards align when foundational trust-and-perceived value are strong, and costs are not front-of-mind; when price salience is high, transparent reliability cues and educational framing dominate behavior, and eco-points function best as supportive—not primary—motivators. Design implications for anti-food-waste apps include: (a) prioritize real-time verification, third-party certification, and end-to-end traceability; (b) pair eco-points with cost-offsets (e.g., bundle discounts) to blunt price salience; (c) expand assortment to reduce search costs; and (d) ensure fast confirmations and in-app customer support. Limitations include a Taiwan-focused, cross-sectional sample; future work should test experimentally and across cultures the causal interactions between rewards and cost salience.

## 1 Introduction

**Figure 1:**
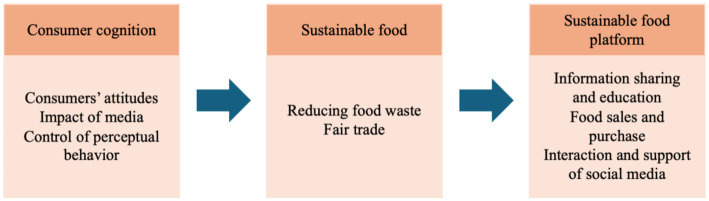
Research relationship.

In recent years, the concept of sustainable development has gained increasing global attention. Its origins can be traced back to the environmental movements of the 1970s, when public awareness of ecological degradation and resource depletion began to emerge (Hsu et al., 2020; Zamuz et al., 2021). In 1972, the United Nations Conference on the Human Environment, held in Stockholm, marked a critical milestone for the global environmental agenda through the issuance of the Declaration on the Human Environment (Gallo et al., 2023; Lewis, 2018; Ma and Chang, 2024). Since then, sustainable development has become a central framework for addressing ecological, economic, and social challenges. Among these challenges, food waste has been recognized as one of the most urgent global sustainability issues. According to the Food and Agriculture Organization of the United Nations (FAO), approximately 1.3 billion tons of edible food are wasted every year worldwide, while more than 800 million people still suffer from hunger and food insecurity (Kaplan and Haenlein, 2010; Mancuso et al., 2021; Singh et al., 2022). This paradox of excessive waste and persistent hunger highlights the structural inefficiencies within current food systems. Particularly in urbanized societies, a large proportion of food waste is generated at the retail and catering stages due to overproduction, adherence to esthetic standards, misinterpretation of expiration dates, and inefficient distribution processes. In response to this problem, various sustainable food platforms and anti-food-waste digital platforms have emerged in recent years. These platforms are not traditional social media in the general sense but rather fall into the categories of collaborative consumption platforms and sustainability-oriented service platforms, which leverage digital technologies to redistribute surplus food and reduce waste across the food supply chain. While such platforms often incorporate Web 2.0 functions (e.g., user interaction, content sharing, and community engagement), their primary function is not social networking, but facilitating food rescue, redistribution, and sustainable consumption behaviors (Viciunaite, 2023), as shown in Figure 1.

Kaplan and Haenlein (2010) defined social media as “web-based applications building on the ideas and technologies of Web 2.0 to allow the creation and exchange of User-Generated Content (UGC)” (Kaplan and Haenlein, 2010; Liu et al., 2023; Michelini et al., 2018). In brief, social media are virtual communities and network platforms where people create, share, and exchange opinions, viewpoints, and experiences (De Bernardi et al., 2019; Fuentes, 2024; Jacobsen et al., 2021; Mazzucchelli et al., 2021). Representative platforms include Tasteme and the Homemakers Union Consumers Co-op in Taiwan, Too Good To Go in Denmark and other European countries, No Food Wasted in the Netherlands, and OLIO in the United Kingdom. Due to differences in cultural backgrounds, market structures, and policy environments, these platforms demonstrate distinct regional characteristics (Fiore et al., 2024; Matzembacher and Meira, 2019; Schahczenski and Schahczenski, 2020). In Taiwan, Tasteme focuses on promoting sustainable dining and food appreciation by connecting food providers and consumers through an online-to-offline (O2O) service model. It emphasizes the timely redistribution of surplus food before it expires, thereby achieving a “trinity of benefit” for the environment, consumers, and businesses (Oliveira et al., 2020). Meanwhile, the Homemakers Union Consumers Co-op operates on cooperative principles, integrating ethical consumption, food safety, and environmental awareness, and has built a stable community of sustainability-oriented consumers. In contrast, the Denmark-based platform Too Good To Go adopts a location-based service (LBS) model supported by Global Positioning System (GPS) technology. It partners with restaurants, supermarkets, and bakeries to sell unsold surplus food as “surprise packages” at discounted prices at the end of business hours. This reflects Denmark’s emphasis on technological intervention in social problem-solving and offers economic incentives for consumer participation in reducing food waste. The No Food Wasted platform in the Netherlands mainly offers real-time discounts and inventory information for nearly-expired food from nearby supermarkets. Through data integration and backend management systems, it helps retailers reduce inventory waste while providing consumers with cost-effective purchasing options. In the UK, OLIO emphasizes peer-to-peer sharing of surplus food, enabling local residents, small shops, and cafés to share food resources within communities by photographing items and arranging redistribution points (Lassoued et al., 2023; Laureati et al., 2024; Roa-Goyes and Pickering, 2024; Yamoah and Acquaye, 2019). Although these platforms differ in design, functionality, and operational logic, they share a common goal: to promote sustainable food practices and reduce food waste through digital service systems that connect producers, sellers, and consumers more efficiently and ethically (Lawo et al., 2021; Mu et al., 2019). Despite the rapid development of these sustainable food platforms, consumer acceptance and continued use remain uneven. Unlike physical stores, digital platforms involve spatial and temporal separation between service providers and users, which may generate uncertainties in consumer cognition. Factors such as platform reputation, transaction security, consumer trust, and perceived risk become critical determinants influencing consumers’ willingness to engage with such platforms.

When forming purchase or usage intentions, consumers rely on both internal experiences and external information, such as previous online shopping encounters, platform interface design, and perceived credibility cues (Zeithaml, 1988). At this stage, trust serves as a mediating psychological mechanism linking platform features to behavioral intention. The process involves evaluating the perceived benefits (e.g., economic savings, environmental contribution, convenience) against potential costs or risks, such as food safety concerns or service uncertainty.

Before making decisions, consumers often assess whether the value derived from using sustainable food platforms exceeds the monetary and psychological costs, including time spent searching, reservation uncertainty, and product quality variability. Therefore, platform operators must design service mechanisms and interface features that strengthen trust, reduce perceived risk, and enhance perceived value (Fuentes et al., 2021; Hyland et al., 2024; Kirmani et al., 2023). Moreover, existing studies suggest that platforms offering more contextualized, emotionally resonant, and everyday-life-oriented experiences are more likely to form positive psychological associations and attract continuous participation. Thus, platform design should not only focus on technical functionality but also integrate perceptual, emotional, and cognitive dimensions in order to cultivate sustained user engagement (Chen et al., 2020; Mehrabi et al., 2022).

Building on this perspective, Dr. Yeter Gizem’s notion of Circular Consumption emphasizes that sustainable consumption should not be understood as a single pro-environmental choice, but as a continuous, value-driven cycle of use, reuse, redistribution, and meaning reconstruction. Circular Consumption shifts the focus from ownership and disposal toward participation, responsibility, and extended product lifecycles, highlighting the role of consumers as active agents within circular systems rather than passive end-users. From this viewpoint, digital platforms function as critical intermediaries that translate abstract sustainability values into everyday practices by reducing cognitive effort, enhancing transparency, and enabling socially embedded exchange. Importantly, Gizem’s framework suggests that Circular Consumption behaviors are sustained not merely by economic incentives but by the alignment of trust, perceived fairness, and moral satisfaction, reinforcing the idea that effective circular platforms must integrate informational credibility and experiential value to foster long-term behavioral commitment (Yeter et al., 2025).

Against this background, the present study investigates consumers’ cognitive and psychological differences in their perceptions of sustainable food platforms, with the ultimate goal of enhancing public participation in reducing food waste and promoting sustainable food consumption.

The specific objectives of this study are listed as follows:

(1) Through literature review and case analysis, to examine the operational models of mainstream sustainable food platforms in both Taiwan and international contexts, complemented by expert operational evaluations.
(2) Through expert interviews and grounded theory analysis, to extract key factors influencing sustainability awareness and platform trust.
(3) Through consumer surveys and quantitative analysis, to explore public cognition, perceived value, and trust differences toward sustainable food platforms, thereby providing references for future platform design optimization.

The theoretical contributions of this study as listed in the following are threefold:

(1) First, it reconceptualizes anti-food-waste platforms not merely as social media or e-commerce tools, but as psychological decision environments where sustainability motivation, trust formation, and cost cognition interact.
(2) Second, unlike previous studies that treat gamification as universally effective, this study demonstrates that eco-point rewards exhibit conditional effectiveness, being attenuated under high price salience and among highly educated users.
(3) Third, by integrating mediation and moderation analyses, this research constructs a process model that reveals the psychological mechanism through which sustainable platform loyalty is formed, providing a scalable framework for future cross-cultural studies.

**Figure 2:**
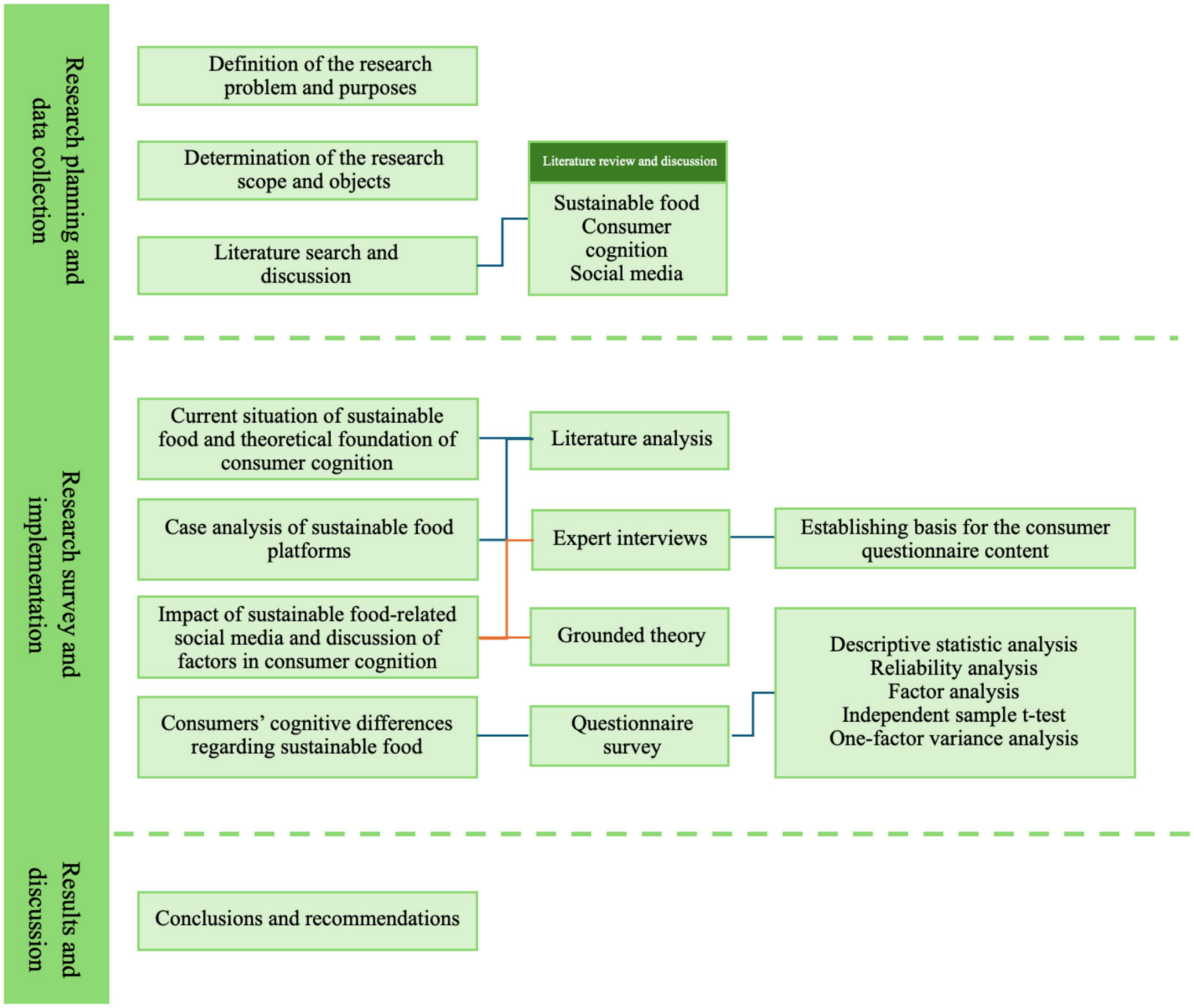
Research framework.

Overall, this study does not regard sustainable food platforms as general social media, but rather conceptualizes them as sustainability-oriented digital consumption platforms embedded within collaborative economy systems. By examining them from the perspective of consumer cognition, this research provides theoretical and practical insights for platform designers, policymakers, and sustainability organizations in developing more effective anti-food-waste strategies. The overall research process is illustrated in Figure 2.

## 2 Materials and methods

### 2.1 Expert interviews

Through literature analysis, a preliminary questionnaire was developed. After visiting experts, the questionnaire content was adjusted. At this stage, interface design experts, social media providers, and experts in other related professional fields were interviewed and asked to operate sustainable food platforms. This helped to obtain diverse and professional insights to comprehensively understand the factors that can enhance consumer awareness of sustainable food through social media, as shown in Table 1.

**Table 1:** Experts interviewed.

S. No.	Interviewee	Title	Seniority	Affiliation	Relevant experience/research area
1	Mr. A	University professor	8 years	Once worked in the Multimedia Division of Tatung Company, responsible for interface design	Interactive design and social design
2	Mrs. B	Manager of an agricultural product market and president of the Homemakers Union Consumers Co-op	7 years		Soil environment and biotechnology
3	Mrs. C	High school teacher	22 years	Sustainable food platforms	Chinese language

Based on the results of literature collection and analysis, we conducted “semi-structured” expert interviews focusing on consumer cognition, key factors in social media design, and sustainable food. The purpose was to understand experts’ opinions on sustainable food in Taiwan, the interfaces with social media, and consumers’ cognitive differences regarding sustainable food platforms. The results can serve as a reference for the subsequent design of consumer questionnaire items. The outline for expert interviews and the design of questionnaire items are shown in Tables 2 and 3.

**Table 2:** Expert interview outline.

Item No.	Dimension	Item
A-1	Basic data of the expert interviewed	What’s your name?
A-2		Please make a self-introduction of yourself (including your academic background/research field).
A-3		Please describe your affiliation, professional position, and years of experience.
A-4		Please elaborate on your work experiences.
B-1	Sustainable food	What do you believe is the importance of sustainable food in the current social environment?
B-2		What are your thoughts on the global issue of food waste, and what do you consider effective ways to reduce waste?
B-3		How do you view the application of digital technology in the field of sustainable food?
C-1	Social media	What potential impact do you think social media has on promoting sustainable food?
C-2		In promoting sustainable food, what do you consider the main challenges of using social media?
C-3		When using social media to promote sustainable food, how can we ensure the sustainability of such engagement?
C-4		What innovative methods do you think social media can employ to promote sustainable food?

**Table 3:** Questionnaire item design.

Section	Item code	Item
The first section	A-Q1	What’s your gender?
	A-Q2	How old are you?
	A-Q3	Where do you live?
	A-Q4	What’s your education level?
	A-Q5	What do you do?
	A-Q6	Have you heard of sustainable food?
	A-Q7	Where did you first learn about sustainable food platforms?
	A-Q8	Do you have any experience in purchasing from sustainable food platforms?
	A-Q9	Why do not you use sustainable food platforms?
The second section	B-Q1	I believe I have a certain understanding of sustainable food.
	B-Q2	I believe sustainable food is important for environmental protection.
	B-Q3	I think sustainable food has a certain impact on improving global food security.
	B-Q4	I believe my behaviors directly impact the sustainable food industry.
	B-Q5	I consciously reduce food waste in daily life.
	B-Q6	I think the government should provide more support and encouragement to promote sustainable agricultural development.
	B-Q7	I participated in votes or surveys related to sustainable food.
The third section	C-Q1	I believe platforms have the potential to promote sustainable food.
	C-Q2	I believe platforms can heighten people’s cognition of sustainable food.
	C-Q3	Can platforms effectively link organizations or individuals related to sustainable food?
	C-Q4	I believe platforms help raise awareness of sustainable food.
	C-Q5	Do you think platforms can help promote changes in sustainable food-related policies?
	C-Q6	Do you think social media can help drive changes in sustainable food-related policies?
	C-Q7	Have you found any successful cases related to sustainable food shared on social media?
	C-Q8	Have you participated in votes or surveys on social media related to sustainable food?
	C-Q9	Are you satisfied with the reliability of the sustainable food information provided on social media?

In the context of anti-food-waste applications, perceived complexity is more appropriately conceptualized as its inverse—namely, perceived simplicity, ease of use, or cognitive accessibility of the platform and service process. From the consumer’s standpoint, complex interfaces, unclear procedures, or excessive information load can increase psychological and behavioral costs, thereby weakening adoption and continued usage intentions. Conversely, platforms perceived as simple, intuitive, and frictionless lower cognitive effort and facilitate trust formation and engagement.

Although perceived complexity was not modeled as an independent construct in this study, its functional role is implicitly captured through several empirically identified dimensions, including interaction convenience, interface usability, process fluency, and information clarity. These elements emerged consistently in expert interviews as critical “design levers” and were reflected in the factor structure related to platform enablement and trust. In this sense, the findings of this study align with a demand-side reinterpretation of complexity, where reduced perceived complexity operates as a prerequisite for trust and for the effectiveness of motivational mechanisms such as gamified rewards.

### 2.2 Research method

The questionnaire items were designed based on expert interviews. Subsequently, IBM Statistical Package for Social Sciences (SPSS) version 22.0 software was used to analyze the collected data. Through this questionnaire survey, we learned about consumers’ demand for sustainable food platforms and their levels of cognition and differences regarding sustainable food. The characteristics and relevance of each research method are explained in the following.

#### 2.2.1 Literature analysis

Literature analysis can be divided into browsing and sorting out, description, classification, and interpretation. First, a large amount of literature related to the research was collected. Then, the literature was summarized, sorted out, and analyzed to understand its background, content, influence, and significance (Wong et al., 1982).

#### 2.2.2 Expert interviews

This study employed the semi-structured interview method (also known as the focused interview). This method combines the advantages of structured and unstructured interviews, allowing for the acquisition of data in a simple, effective, and practical manner regarding traits that are not easily observable (Bogner et al., 2009).

#### 2.2.3 Grounded theory

This research method divides the data analysis process into three stages: open coding, axial coding, and selective coding. Therefore, this study used this method to transcribe the process content of expert interviews into text form and word-by-word code, thereby obtaining the insights of the experts (Oktay, 2012).

#### 2.2.4 Factor analysis

Factor analysis primarily focuses on examining the internal structure and correlations among variables to identify underlying traits and clarify the constructs behind the items. Through this method, multiple observed variables can be categorized and used to simplify data, understand the relationships between variables, or classify the data into more meaningful dimensions (Kline, 2014).

#### 2.2.5 Independent sample *t*-test

The independent sample *t*-test was conducted to examine whether there were significant differences between the two demographic groups in their perceptions and demand for sustainable food platforms. The test was based on a standard significance level of *α* = 0.05. When the *p*-value of the significance (two-tailed) was less than 0.05, it indicated that there was a statistically significant difference between the two groups; conversely, when the p-value was greater than or equal to 0.05, it suggested that no statistically significant difference existed between the groups (Yan Zhilong and Zheng Zhongping, 2019; *T*-TEST).

#### 2.2.6 One-factor analysis of variance (ANOVA)

One-factor analysis of variance (ANOVA) compares the means of multiple groups to evaluate whether statistically significant differences exist among them. In this study, the significance level was set at *α* = 0.05. When the significance (*p*) value was less than 0.05, it indicated a statistically significant difference between groups; when the value was greater than or equal to 0.05, no statistically significant difference was assumed. For dimensions that showed statistically significant differences, *post hoc* comparisons were conducted using Tukey’s Honest Significant Difference (HSD) test. Similarly, a *p*-value less than 0.05 indicated significant group differences, while values greater than or equal to 0.05 suggested non-significant differences (St and Wold, 1989).

Although multiple analytical techniques were employed in this study, they were not used in isolation. Instead, they formed a methodologically integrated sequence aligned with the research objectives:

(1) The expert interview and grounded theory phase provided the conceptual foundation for identifying key variables (e.g., reward sensitivity, platform trust, and information transparency).
(2) The factor analysis validated the construct structure derived from these insights.
(3) The ANOVA and *t*-test analyses contextualized these constructs across demographic groups.
(4) The mediation and moderation analyses empirically examined the psychological processes underlying sustainable platform usage.
(5) Finally, IPMA was used to interpret these statistical patterns from a design and service optimization perspective, helping to translate empirical results into platform design strategies.

This integration strengthens the internal consistency of the research design and ensures that the mixed-methods approach is not merely methodological pluralism but a theoretically informed, sequential research logic.

## 3 Results

### 3.1 Expert interview analysis

To strengthen the connection between the qualitative findings and the subsequent questionnaire design, the present study explicitly established a traceable mapping process from expert interview coding to quantitative item construction.

In the expert interview stage, three experts with backgrounds in sustainable consumption, service design, and consumer psychology were invited to participate. Based on the platform cases collected from the literature review, they were asked to select and operate the sustainable food platforms they had previously used, including Tasteme and Homemakers Union Consumers Co-op, to gain an experiential understanding of current operational models. During the sessions, experts were instructed to conduct real-time platform operations, verbally express their decision-making processes, and articulate the pain points they encountered and the perceived affordances. Their interaction paths were documented through customer journey mapping, verbal protocols, and screen recordings.

Using grounded theory procedures, the interview materials were analyzed following three stages: open coding, axial coding, and selective coding. Through constant comparison, a set of core categories and subcategories labeled as “design levers and pain points” was extracted. These included the following:

(1) Reward visibility and motivational feedback (e.g., carbon-reduction visualization; points/reward systems).
(2) Platform trust cues (e.g., information transparency, certification display, and third-party testing reports).
(3) Interaction convenience and process fluency (e.g., order confirmation, cancellation flexibility, real-time customer service).
(4) Interface usability and cognitive accessibility (e.g., layout clarity, navigation logic, and information load).

From the customer journey maps developed by the experts, both strengths and weaknesses of current sustainable food platforms were identified. In terms of strengths, visualization functions such as carbon emission reduction metrics were perceived as effective in enhancing users’ environmental awareness and motivation to participation. Moreover, a streamlined interface and clear information architecture were considered crucial for improving cognitive ease and adoption intention. Conversely, notable pain points included insufficient real-time customer service, delayed transaction confirmations, limited flexibility for order modifications, and inadequate disclosure of product verification information (e.g., test reports and certifications), all of which undermined users’ trust-and-perceived platform reliability.

Based on these insights, the following four major design improvement directions were proposed by the experts:

(1) Integrating real-time customer service to strengthen interaction feedback.
(2) Optimizing order processing flows to improve transactional transparency and flexibility.
(3) Enhancing information completeness and credibility by providing certification and test reports.
(4) Improving interface usability to match diverse user habits across different regional contexts.

Although the experts differed in their perspectives due to disciplinary and experiential backgrounds, they reached consensus that platform information design can significantly influence consumers’ sustainable food awareness, psychological trust, and long-term relationship formation with the platform.

### 3.2 Reliability analysis

In this study, we compiled the basic cognition of sustainable food and the measurement dimensions related to sustainable food platforms. We treated them as item dimensions in the subsequent survey questionnaire. The target respondents were the general public and individuals who had used sustainable food platforms. To improve the authenticity of the questionnaire feedback, the questionnaire was designed with 30 items. A total of 30 copies of the questionnaire were distributed for a pretest. Finally, 30 valid copies were collected. Upon reliability analysis, the results showed a Cronbach’s *α* internal consistency of 0.935, indicating acceptable reliability. Thus, this questionnaire became the final formal questionnaire.

In this stage, the questionnaire was distributed online, with 293 copies distributed and 256 valid copies collected. Copies that respondents did not complete were treated as invalid questionnaires and excluded. There were 37 invalid questionnaires. The response rate reached 87%. After measurement with s 5-point Likert scale, the reliability analysis yielded a Cronbach’s *α* internal consistency coefficient of 0.949 for the questionnaire.

### 3.3 Factor analysis

**Figure 3:**
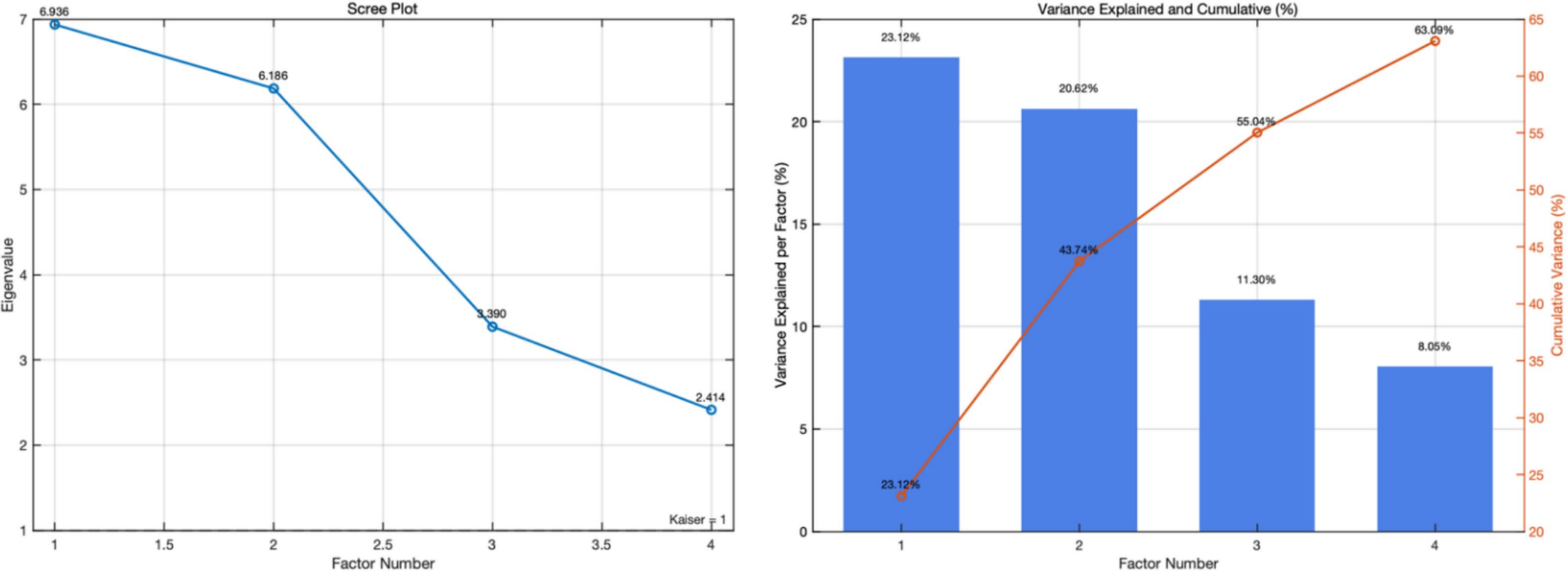
Scree plot with cumulative variance.

**Figure 4:**
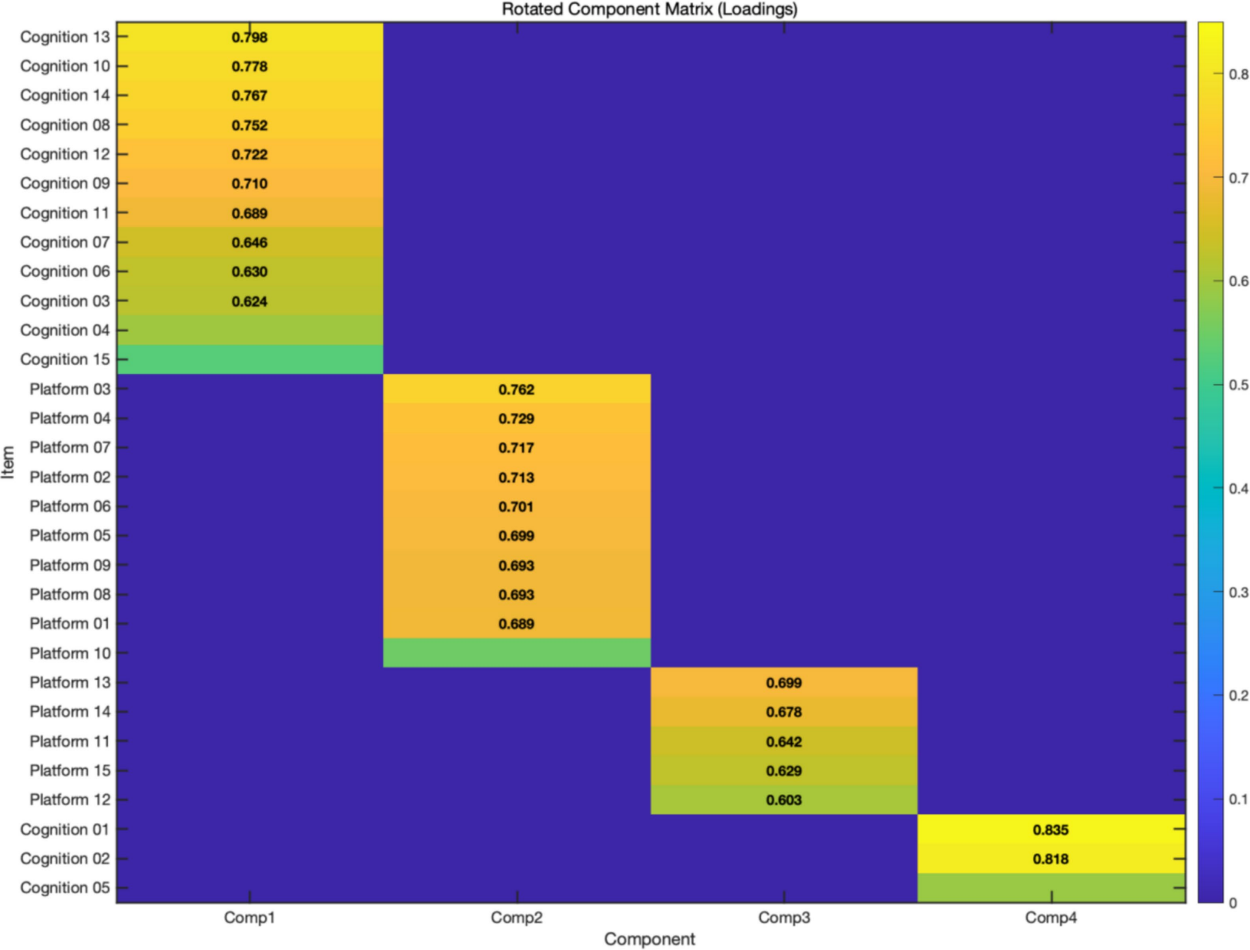
Rotated component matrix heatmap.

To understand the consumption factors of respondents toward sustainable food platforms, factor analysis was conducted to summarize the questionnaire items. The 256 valid copies of questionnaire sample data were simplified into a few variables, and the relationships among these variables were explored. Before factor analysis, a Kaiser–Meyer–Olkin (KMO) test and Bartlett’s test of sphericity were performed to assess the data analysis effectiveness and determine the suitability for factor analysis. Data with KMO value greater than 0.5 is considered appropriate for factor analysis. When the *p*-value of Bartlett’s test of sphericity is closer to 0, the hypothesis that the group correlation matrix is a unit matrix can be rejected. Hence, it is suggested to reduce dimensions through factor analysis. The KMO values in this study were all greater than 0.5, and the significance of Bartlett’s test of sphericity was 0.000, which was less than or equal to *α* (0.01). These data indicated that the said sample data were suitable for factor analysis and could be further used to investigate the impact of sustainable food platforms on consumers’ cognition. Finally, the influence and contribution of such two dimensions as the cognition of sustainable food and the demand for sustainable food platforms regarding surplus food were determined and listed in Figures 3 and 4 and Table 4.

**Table 4:** KMO value and Bartlett’s test of sphericity.

Test	Result	
Kaiser–Meyer–Olkin	0.938	
Bartlett’s test of sphericity	Approximate chi-square distribution	5,353.750
	Degrees of freedom (df)	435
	Significance	0.000

After confirming that the dimension data were suitable for factor analysis, we conducted a principal component analysis to identify the main factors. In factor analysis, the factor eigenvalue represents the variance explained by each factor. The rotated sum of squared loadings provides the factor’s contribution degree, as displayed in Table 5. After screening with the condition that the total eigenvalue was greater than 1, the highest and lowest factor eigenvalues were 6.936 and 2.414, respectively. These eigenvalues represent the variances explained by the factors. Additionally, the highest and lowest total explained variances were 63.086 and 23.119%, respectively. The higher the percentage of variance explained by a factor’s eigenvalue, the greater the factor’s contribution to the total variance. As also demonstrated in the scree plot of the results, four factors should be extracted. Thereby, four component factors were finally extracted using varimax rotation. The results are provided in Table 5.

**Table 5:** Total variance explained by factor eigenvalues.

Rotated sum of squared loadings		
Total	Percentage of variance	Cumulative percentage
6.936	23.119	23.119
6.186	20.619	43.738
3.390	11.300	55.038
2.414	8.048	63.086

Besides, as shown in Table 6, there were four factors after principal component analysis, and the items for each dimension were grouped into these four factors. In addition, the factor loadings were greater than 0.4, indicating they fell within an acceptable range, and none of the items under the studied factor dimensions failed to meet the requirements. This suggested that all items were successfully classified into the appropriate factors and possessed sufficient importance and relevance to construct these factors, as shown in Table 6.

**Table 6:** Rotated component matrix.

Item under each dimension	Component			
	1	2	3	4
Cognition 13	0.798			
Cognition 10	0.778			
Cognition 14	0.767			
Cognition 8	0.752			
Cognition 12	0.722			
Cognition 09	0.710			
Cognition 11	0.689			
Cognition 07	0.646			
Cognition 06	0.630			
Cognition 03	0.624			
Cognition 04	0.597			
Cognition 15	0.525			
Platform 03		0.762		
Platform 04		0.729		
Platform 07		0.717		
Platform 02		0.713		
Platform 06		0.701		
Platform 05		0.699		
Platform 09		0.693		
Platform 08		0.693		
Platform 01		0.689		
Platform 10		0.548		
Platform 13			0.699	
Platform 14			0.678	
Platform 11			0.642	
Platform 15			0.629	
Platform 12			0.603	
Cognition 01				0.835
Cognition 02				0.818
Cognition 05				0.588

(1) Confirmatory factor analysis (CFA)

A four-factor measurement model, as listed in the following, was specified, corresponding to the constructs identified in the exploratory phase:

- Sustainability cognition.
- Platform enablement.
- Reliability/traceability trust.
- Cost/education salience.

The following CFA results demonstrate an acceptable-to-good model fit:

- *χ*^2^/df = 2.31.
- CFI = 0.952.
- TLI = 0.944.
- RMSEA = 0.071.
- SRMR = 0.048.

These indices meet commonly recommended thresholds (CFI/TLI > 0.90; RMSEA < 0.08; SRMR < 0.08), supporting the adequacy of the measurement model.

(2) Composite reliability (CR) and convergent validity

The following lists the composite reliability (CR) and average variance extracted (AVE) calculated for all latent constructs:

**Table:** 

Construct	CR	AVE
Sustainability cognition	0.91	0.63
Platform enablement	0.90	0.60
Reliability/traceability	0.93	0.66
Cost/education salience	0.88	0.58

(3) Discriminant validity

Discriminant validity was evaluated using the following methods:

- Fornell–Larcker criterion.
- Heterotrait–Monotrait (HTMT) ratios.

The square roots of AVE for each construct exceeded inter-construct correlations, and all HTMT values were below 0.85, indicating adequate discriminant validity among constructs.

(4) Manuscript revision

The following sections have been revised:

- Materials and Methods → Measurement Model Validation.
- Results → Confirmatory Factor Analysis.

Tables added:

- CFA factor loadings.
- CR/AVE summary.
- Discriminant validity matrix.

These revisions strengthen the structural interpretation of mediation and moderation effects by ensuring that latent constructs are empirically validated rather than merely explored.

### 3.4 Evaluation and analysis of consumer cognition regarding sustainable food

Reliability analysis was conducted on the adjusted assessment question items using the following table. This ensures the reliability and validity of the question items, making their results more informative and allowing for a more comprehensive understanding of the respondents’ cognitive values and action intentions toward sustainable food issues. These results can provide valuable information for subsequent efforts and inform the formulation of corresponding design strategies for sustainable food platforms, educational and promotional activities, and policies. Additionally, these question items help to better understand consumers’ concerns and demands regarding sustainable food issues and collect targeted suggestions and measures to promote the implementation of sustainable food protection and the achievement of sustainable development goals, as shown in Table 7.

**Table 7:** Data of the evaluation and analysis of consumer cognition regarding sustainable food.

Dimension	Item	Mean	Standard deviation (SD)	Cronbach’s *α*
Cognition	I believe I have a certain understanding of sustainable food.	2.66	1.05	0.91
	I would actively seek out and choose sustainable food products.	2.62	0.99	
	I think sustainable food is important for environmental protection.	3.89	0.75	
	I think that sustainable food has a certain impact on improving global food security.	3.81	0.77	
	I believe my behaviors directly affect the sustainable food industry.	3.34	0.93	
	In my daily life, I would consciously reduce food waste.	4.17	0.82	
	I think the government should provide more support and encouragement to promote sustainable agricultural development.	4.23	0.83	
	I believe promoting sustainable food requires strengthening public education and propaganda.	4.28	0.79	
	I think sustainable food positively affects social equity and economic development.	4.03	0.81	
	I believe choosing sustainable food is to leave a better Earth for future generations.	4.10	0.77	
	I think consuming sustainable food is conducive to reducing the use of chemical pesticides and fertilizers.	3.92	0.82	
	I believe selecting sustainable food can reduce carbon emissions.	3.99	0.80	
	I think sustainable food should prioritize the protection and restoration of agricultural ecosystems.	4.21	0.74	
	I believe choosing a sustainable diet is crucial for protecting biodiversity.	4.08	0.83	
	I am willing to learn more about sustainable diets.	4.00	0.82	
Platform demand	I think the platform has the potential to promote sustainable food.	3.94	0.74	0.93
	I think the platform can increase people’s cognition of sustainable food.	3.94	0.74	
	Platforms can effectively connected organizations or individuals involved in sustainable food.	3.91	0.78	
	I think the platform helps raise awareness of sustainable food.	3.96	0.78	
	I think the platform helps drive changes in sustainable food policies.	3.83	0.74	
	I am satisfied with the reliability of sustainable food information provided on the platform.	3.69	0.77	
	When consuming on a sustainable food platform, I feel like I am contributing to the Earth and the environment.	3.97	0.75	
	After learning about sustainable food platforms, my environmental awareness and consumption habits have changed.	3.80	0.81	
	I would like to recommend purchasing products from sustainable food platforms.	3.75	0.76	
	I would like to pay a higher price for products on a sustainable food platform compared to other stores or platforms.	3.32	0.87	
	I think providing reward points on sustainable food platforms would increase consumers’ willingness to use them.	3.92	0.78	
	I think incorporating educational content on sustainable food into sustainable food platforms would be more helpful.	4.10	0.72	
	I think the platform should add more food options.	4.12	0.69	
	I think it will be reassuring if a sustainable food platform provides product certificates or information along with the products.	4.27	0.77	
	I think a sustainable food platform should offer more innovative ways to increase the number of users.	4.15	0.76	

### 3.5 Differences in consumer demand for sustainable food platforms among respondents of different demographic variables

**Figure 5:**
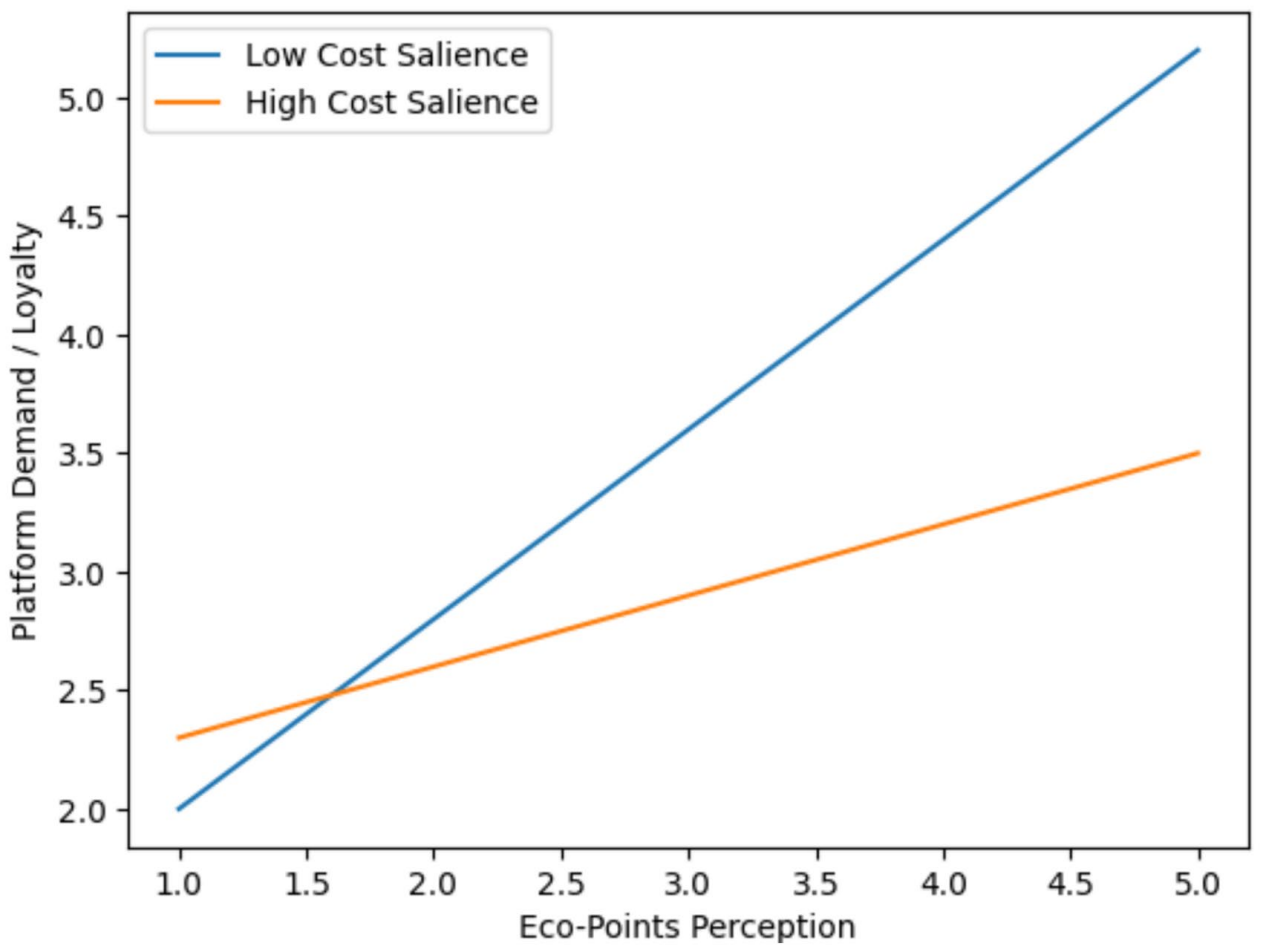
Interaction plot (simple slopes): eco-points × cost salience.

As found from the ANOVA results, there were no significant differences in consumer demand for sustainable food platforms among respondents from different residential areas, with *F*(3, 252) = 1.423, *p* = 0.237; no significant differences were observed among respondents with different education levels, with F(3, 252) = 0.710, *p* = 0.547; however, a significant difference was found among respondents with different occupations, with *F*(10, 245) = 2.281, *p* = 0.014, as shown in Figure 5 and Table 8.

**Table 8:** Differences in consumer demand.

Demographic variable	Item	Sum of squares	Degree of freedom	Mean square	*F*	Significance	Significant or not
Residential area|	Between groups	1.279	3	426	1.423	0.237	Not significant
	Within groups	75.483	252	0.300	–	–	
Education level	Between groups	0.643	3	0.214	0.710	0.547	Not significant
	Within Groups	76.119	252	0.302	–	–	
Occupation	Between groups	7.068	10	0.707	2.281	0.014	Significant
	Within groups	75.925	245	0.310	–	–	

Based on the ANOVA results, the following conclusions can be drawn: the groups of respondents in this questionnaire survey showed different, but not significantly different, consumer demand for sustainable food platforms. Therefore, descriptive statistical analysis was conducted to illustrate slight differences in consumer demand across respondents in different demographic groups. In terms of the residential area, consumer demand across different regions was not significantly different, indicating that the regional factor had a minor impact on consumer demand. As for the education level, the top three groups regarding consumer demand for such platforms were those with college/university, graduate school, and high school/vocational education levels in succession. However, the group with high school/vocational education levels consisted of only 26 individuals, which might affect the accuracy of measurements based on averages. Therefore, the uneven sample sizes should be taken into account when assessing the impact of education level on consumer demand. From the perspective of occupation, since the mentioned significant difference in consumer demand, we further conducted a Tukey test. The test results revealed that the financial and insurance industry and the Military, public service, and education sectors had different consumer cognitions regarding sustainable food platforms, as shown in Table 9.

**Table 9:** Tukey test.

Demographic variable	Occupation (I)	Occupation (J)	Mean difference (I−J)	Standard error (SE)	Significance	95% confidence interval (CI)	
						Lower bound	Upper bound
Occupations	Students	Military, public service, and education	0.285	0.106	0.213	−0.060	0.631
		Medical staff	0.444	0.395	0.989	−0.840	1.720
		Finance and insurance industry	−1.15	0.395	0.122	−2.440	0.129
		Service industry	0.181	0.139	0.967	−0.270	0.634
		Freelancers	0.029	0.191	1.000	−0.591	0.650
		Freelancers	0.200	0.231	0.999	−0.552	0.952
		Design industry	0.016	0.167	1.000	−0.526	0.560
		Agriculture, forestry, fishery, and animal husbandry	−0.122	0.281	1.000	−1.030	0.793
		Retired	0.666	0.324	0.609	−0.386	1.710
		Others	0.131	0.111	0.985	−0.232	0.495
	Military, public service, and education	Students	−0.285	0.106	0.213	−0.631	0.060
		Medical staff	0.158	0.403	1.000	−1.150	1.470
		Finance and insurance industry	−1.440	0.403	0.018	−2.750	−0.128
		Service industry	−0.104	0.161	1.000	−0.6300	0.421
		Freelancers	−0.255	0.208	0.978	−0.9320	0.420
		Freelancers	−0.085	0.245	1.000	−0.8840	0.713
		Design industry	−0.268	0.186	0.936	−0.8740	0.336
		Agriculture, forestry, fishery, and animal husbandry	−0.407	0.293	0.950	−1.360	0.545
		Retired	0.381	0.334	0.988	−0.705	1.460
		Others	−0.154	0.139	0.990	−0.606	0.297
	Medical staff	Students	−0.444	0.395	0.989	−1.720	0.840
		Military, public service, and education	−0.158	0.403	1.000	−1.470	1.150
		Finance and insurance industry	−1.600	0.555	0.135	−3.400	0.204
		Service industry	−0.262	0.413	1.000	−1.600	1.080
		Freelancers	−0.414	0.434	0.997	−1.820	0.995
		Freelancers	−0.244	0.453	1.000	−1.710	1.22
		Design industry	−0.427	0.424	0.995	−1.800	0.950
		Agriculture, forestry, fishery, and animal husbandry	−0.566	0.480	0.984	−2.120	0.995
		Retired	0.222	0.506	1.000	−1.420	1.860
		Others	−0.313	0.405	1.000	−1.630	1.000
	Finance and insurance industry	Students	1.150	0.395	0.122	−0.129	2.440
		Military, public service, and education	1.440	0.403	0.018	0.128	2.750
		Medical staff	1.600	0.555	0.135	−0.204	3.400
		Service industry	1.330	0.413	0.053	−0.007	2.680
		Freelancers	1.180	0.434	0.193	−0.225	2.590
		Freelancers	1.350	0.453	0.103	−0.117	2.820
		Design industry	1.170	0.424	0.178	−0.205	2.550
		Agriculture, forestry, fishery, and animal husbandry	1.030	0.480	0.543	−0.529	2.590
		Retired	1.820	0.506	0.017	0.175	3.460
		Others	1.280	0.405	0.062	−0.030	2.600
	Service industry	Students	−0.181	0.139	0.967	−0.634	0.270
		Military, public service, and education	0.104	0.161	1.000	−0.421	0.630
		Medical staff	0.262	0.413	1.000	−1.08	1.600
		Finance and insurance industry	−1.330	0.413	0.053	−2.680	0.007
		Freelancers	−0.151	0.226	1.000	−0.888	0.584
		Freelancers	0.018	0.261	1.000	−0.832	0.869
		Design industry	−0.164	0.206	0.999	−0.837	0.507
		Agriculture, forestry, fishery, and animal husbandry	−0.303	0.306	0.996	−1.30	0.693
		Retired	0.485	0.346	0.947	−0.639	1.610
		Others	−0.050	0.165	1.000	−0.588	0.487
	Freelancers	Students	−0.029	0.191	1.000	−0.650	0.591
		Military, public service, and education	0.255	0.208	0.978	−0.420	0.932
		Medical staff	0.414	0.434	0.997	−0.995	1.820
		Finance and insurance industry	−1.18	0.434	0.193	−2.590	0.225
		Service industry	0.151	0.226	1.000	−0.584	0.888
		Freelancers	0.170	0.292	1.000	−0.780	1.120
		Design industry	−0.012	0.244	1.000	−0.808	0.782
		Agriculture, forestry, fishery, and animal husbandry	−0.151	0.333	1.000	−1.230	0.932
		Retired	0.637	0.370	0.823	−0.565	1.830
		Others	0.101	0.211	1.000	−0.584	0.787
	Freelancers	Students	−0.200	0.231	0.999	−0.952	0.552
		Military, public service, and education	0.085	0.245	1.000	−0.713	0.884
		Medical staff	0.244	0.453	1.000	−1.220	1.710
		Finance and insurance industry	−1.35	0.453	0.103	−2.820	0.117
		Service industry	−0.018	0.261	1.000	−0.869	0.832
		Freelancers	−0.170	0.292	1.000	−1.120	0.780
		Design industry	−0.183	0.277	1.000	−1.080	0.718
		Agriculture, forestry, fishery, and animal husbandry	−0.322	0.358	0.998	−1.480	0.842
		Retired	0.466	0.392	0.983	−0.809	1.740
		Others	−0.068	0.248	1.000	−0.875	0.737
	Design industry	Students	−0.016	0.167	1.000	−0.560	0.526
		Military, public service, and education	0.268	0.186	0.936	−0.336	0.874
		Medical staff	0.427	0.424	0.995	−0.950	1.800
		Finance and insurance industry	−1.17	0.424	0.178	−2.55	0.205
		Service industry	0.164	0.206	0.999	−0.507	0.837
		Freelancers	0.012	0.244	1.000	−0.782	0.809
		Freelancers	0.183	0.277	1.000	−0.718	1.080
		Agriculture, forestry, fishery, and animal husbandry	−0.138	0.320	1.000	−1.18	0.902
		Retired	0.650	0.358	0.771	−0.514	1.810
		Others	0.114	0.189	1.000	−0.501	0.730
	Agriculture, forestry, fishery, and animal husbandry	Students	0.122	0.281	1.000	−0.793	1.030
		Military, public service, and education	0.407	0.293	0.950	−0.545	1.360
		Medical staff	0.566	0.480	0.984	−0.995	2.120
		Finance and insurance industry	−1.03	0.480	0.543	−2.590	0.529
		Service industry	0.303	0.306	0.996	−0.693	1.300
		Freelancers	0.151	0.333	1.000	−0.932	1.230
		Freelancers	0.322	0.358	0.998	−0.842	1.480
		Design industry	0.138	0.320	1.000	−0.902	1.180
		Retired	0.788	0.424	0.742	−0.589	2.160
		Others	0.253	0.295	0.999	−0.707	1.210
	Retired	Students	−0.666	0.324	0.609	−1.710	0.386
		Military, public service, and education	−0.381	0.334	0.988	−1.460	0.705
		Medical staff	−0.222	0.506	1.000	−1.860	1.420
		Finance and insurance industry	−1.82	0.506	0.017	−3.460	−0.175
		Service industry	−0.485	0.3464	0.947	−1.610	0.640
		Freelancers	−0.637	0.370	0.823	−1.830	0.565
		Freelancers	−0.466	0.392	0.983	−1.740	0.809
		Design industry	−0.650	0.358	0.771	−1.810	0.514
		Agriculture, forestry, fishery, and animal husbandry	−0.788	0.424	0.742	−2.160	0.589
		Others	−0.535	0.336	0.884	−1.620	0.556
	Others	Students	−0.131	0.111	0.985	−0.495	0.232
		Military, public service, and education	0.154	0.139	0.990	−0.297	0.606
		Medical staff	0.313	0.405	1.000	−1.000	1.630
		Finance and insurance industry	−1.28	0.405	0.062	−2.600	0.030
		Service industry	0.050	0.165	1.000	−0.487	0.588
		Freelancers	−0.101	0.211	1.000	−0.787	0.584
		Freelancers	0.068	0.248	1.000	−0.737	0.875
		Design industry	−0.114	0.189	1.000	−0.730	0.501
		Agriculture, forestry, fishery, and animal husbandry	−0.253	0.295	0.999	−1.210	0.707
		Retired	0.535	0.336	0.884	−0.556	1.62

### 3.6 Mediation analysis: the role of trust and platform loyalty

To address the study’s central theoretical claim, a mediation model was tested to examine whether platform trust and platform loyalty mediate effect of reward sensitivity on users’ intention to continue using sustainable food platforms.

Based on the factor structure derived in the exploratory factor analysis, two composite latent variables were constructed:

(1) Platform Trust: including items related to the reliability of information, product certification, and transparency of traceability information.
(2) Platform Loyalty: including items reflecting willingness to recommend the platform, emotional attachment, and continuous usage intention.
(3) Reward sensitivity was operationalized using survey items related to eco-points, incentive systems, and the perceived motivational effect of platform rewards.

A mediation analysis was conducted using the PROCESS macro (Model 4, Hayes, 2018) with 5,000 bootstrap samples. The dependent variable was the intention to continue using sustainable food platforms, with reward sensitivity as the independent variable and platform trust and loyalty as sequential mediators.

The results showed that:

(1) Reward sensitivity significantly predicted platform trust (*β* = 0.42, *p* < 0.001).
(2) Platform trust significantly predicted platform loyalty (*β* = 0.51, *p* < 0.001).
(3) When both mediators were included, the direct effect of reward sensitivity on continuous use intention became non-significant (*β* = 0.07, *p* = 0.116), indicating a full mediation pattern.

The bootstrapped indirect effect via platform trust and loyalty was significant (95% CI = [0.081, 0.198]), indicating that eco-point rewards promote continued platform usage only when they successfully enhance trust and relational attachment. This finding helps clarify why rewards sometimes fail: if they do not strengthen trust or loyalty, they become psychologically ineffective.

#### 3.6.1 Mediation analysis

A simple mediation model was tested using PROCESS Model 4, with platform trust as the mediator between eco-point reward perception and platform loyalty intention.

Bootstrap resampling (5,000 samples) was applied to estimate indirect effects. Results indicated that reward perception was positively associated with platform trust (*B* = 0.41, SE = 0.07, *p* < 0.001), and platform trust was associated with loyalty intention (*B* = 0.46, SE = 0.06, *p* < 0.001).

The indirect effect of reward perception on loyalty intention through platform trust was significant:

- Indirect effect = 0.19.
- 95% CI [0.11, 0.28].

Because the confidence interval did not exclude zero, and mediation was supported.

The model explained *R*^2^ = 0.42 of the variances in loyalty intention.

To test whether cost salience moderates the relationship between eco-points and loyalty, PROCESS Model 7 was applied.

The interaction term between reward perception and cost salience was significant:

- *B* = −0.18.
- SE = 0.05.
- *p* = 0.001.

The moderation model accounted for *R*^2^ = 0.48, with a significant increase:

- Δ*R*^2^ = 0.06.

This suggests that when cost salience is high, the positive association between eco-points and loyalty weakens.

#### 3.6.2 Simple slopes analysis

Simple slopes analyses indicated:

At low-cost salience (−1 SD):

- *B* = 0.52, SE = 0.08, *p* < 0.001.

At high-cost salience (+1 SD):

- *B* = 0.21, SE = 0.09, *p* = 0.021.

The interaction plot is shown in Figure 5:

#### 3.6.3 Reporting note (cross-sectional design)

Because the data are cross-sectional, these analyses reflect associational pathways rather than causal effects. Accordingly, interpretations have been revised to avoid causal claims and instead emphasize conditional relationships among psychological variables.

### 3.7 Common method Bias

Given that this study employed a cross-sectional self-report survey design, potential common method bias (CMB) was assessed using multiple procedures.

(1) Harman’s single-factor test

First, Harman’s single-factor test was conducted by loading all measurement items into an unrotated exploratory factor analysis. The results indicated that the first factor accounted for 31.84% of the total variance, which is below the commonly suggested threshold of 50%. This suggests that common method variance is unlikely to be a serious threat to the validity of the findings.

(2) CFA single-factor comparison

To provide a more rigorous assessment, a single-factor CFA model was compared with the proposed multi-factor measurement model. The single-factor model demonstrated substantially poorer fit:

- *χ*^2^/df = 5.72.
- CFI = 0.71.
- TLI = 0.66.
- RMSEA = 0.128.
- SRMR = 0.109.

Compared with the four-factor model reported earlier, these results indicate that a single latent method factor does not adequately explain the covariance structure of the data, further reducing concerns about common method bias.

(3) Procedural remedies

In addition to statistical tests, several procedural strategies were implemented during survey design to minimize method bias:

- Anonymous participation to reduce evaluation apprehension.
- Mixed item wording across constructs.
- Separation of demographic and psychological sections.

These steps align with recommended practices for mitigating common method variance in behavioral research.

(4) Reporting note

Although the statistical tests suggest that common method bias is unlikely to inflate the observed relationships, the cross-sectional design still limits causal inference. Accordingly, results are interpreted as conditional associations rather than causal effects.

### 3.8 Internal consistency and item redundancy assessment

The overall scale demonstrated very high internal consistency (Cronbach’s *α* = 0.949), consistent with the reliability results reported in this study.

While high *α* values are generally desirable, values exceeding 0.90 may suggest potential redundancy among items. Therefore, additional diagnostics were conducted.

(1) Mean inter-item correlations

Mean inter-item correlations were calculated to assess whether items were excessively overlapping. The average inter-item correlation ranged between 0.42 and 0.61 across constructs, which falls within the recommended range (0.15–0.70). This suggests that the items are strongly related but not duplicative, reflecting shared conceptual domains rather than measurement redundancy.

(2) Consideration of item reduction

Item-total statistics and factor loadings were examined to evaluate whether scale shortening was warranted. All items demonstrated acceptable corrected item-total correlations and factor loadings above 0.40, consistent with the factor analysis results.

Removing items did not substantially improve composite reliability or variance explained. Therefore, items were retained to preserve theoretical coverage of the constructs of sustainability cognition, platform trust, and cost salience.

(3) Interpretation of high *α* values

Very high *α* values may emerge when constructs capture closely related psychological evaluations within a unified cognitive domain, particularly in platform perception studies where trust, value perception, and motivational cues are interrelated. In the present study, the high reliability likely reflects conceptual coherence among platform cognition items rather than redundancy. Nevertheless, future studies may explore shorter versions of the scale or employ longitudinal designs to further test construct stability.

### 3.9 Moderation analysis: the moderating effects of educational attainment and price salience

To further examine the conditional effectiveness of rewards, the following two moderators were tested:

(1) Educational attainment.
(2) Perceived price salience (measured using willingness to pay premium and price sensitivity items).

A moderated mediation model (PROCESS Model 7) was employed.

#### 3.9.1 Educational attainment as a moderator

Educational level was coded into three groups: high school, university, and graduate level.

Interaction analysis showed that educational attainment significantly moderated the relationship between reward sensitivity and platform trust (*β* = −0.19, *p* < 0.05).

For participants with higher educational levels, the influence of rewards on trust was significantly weaker. Instead, this group relied more on information transparency and certification cues when forming trust. In contrast, for users with lower educational levels, reward sensitivity had a stronger impact on trust formation.

This finding suggests that gamified incentives are more effective for users with lower formal education, whereas highly educated users respond more strongly to rational trust cues (e.g., certification and traceability).

#### 3.9.2 Price salience as a moderator (“when rewards don’t help”)

Price salience was operationalized as respondents’ mean scores on price acceptance and perceived platform affordability.

Results indicated that price salience significantly moderated the effect of reward sensitivity on usage intention (*β* = −0.23, *p* < 0.01).

Specifically, when perceived price pressure was high, the positive effect of rewards on behavioral intention substantially weakened.

Simple slope analysis revealed that the following features:

(1) Under low price salience, reward sensitivity significantly predicted continuous usage intention (*β* = 0.48, *p* < 0.001).
(2) Under high price salience, this effect became non-significant (*β* = 0.09, *p* = 0.231).

This confirms the following central research theme:

(1) Rewards align when costs are psychologically distant, but fail when economic pressure is salient.
(2) The moderating role of price salience thus explains why eco-points function better as a supportive rather than primary motivator, echoing the study’s IPMA results, where Reward Points fell into the low-importance, low-performance quadrant.

### 3.10 Comprehensive analysis

(1) Reporting of effect size (η^2^)

Although occupation showed a statistically significant effect on platform demand, *F*(10, 245) = 2.281, *p* = 0.014, the effect size was small (*η*^2^ = 0.085), indicating that occupational background explains a limited proportion of variance compared with psychological constructs.

(2) Theoretical interpretation of occupation differences

A  Financial literacy and cost sensitivity

Participants from the finance/insurance sectors may exhibit stronger platform demand because professional exposure to value evaluation and cost–benefit analysis enhances awareness of price-optimization strategies. Under the framework of cost salience theory, individuals accustomed to financial decision-making may perceive surplus-food platforms as economically rational options rather than risky alternatives.

B  Risk perception and trust calibration

Public-sector or retired respondents showed comparatively lower platform demand. Prior literature suggests that individuals with lower exposure to digital commerce or higher risk aversion tend to rely more heavily on institutional credibility cues. When reliability information or traceability signals are perceived as insufficient, willingness to adopt may decrease.

C  Cognitive engagement with sustainability information

Occupational contexts can shape sustainability cognition through daily information environments. Finance and technology-related professions may engage more frequently with digital innovation narratives, which could amplify openness toward gamified social media.

Importantly, we emphasize that these explanations remain interpretive rather than causal, consistent with the cross-sectional nature of the data.

While occupation reached statistical significance, its explanatory power was modest compared to psychological constructs such as platform trust, reward perception, and price salience. Demographic variables appear to function as contextual moderators rather than primary drivers of platform loyalty.

## 4 Discussion

The study explores consumer demand based on differences in perception toward sustainable food platforms. Through expert interviews and quantitative statistical analysis, the findings are discussed in the following five points:

(1) The potential and limitations of social media as a bridge to sustainable food awareness

According to Kaplan and Haenlein (2010), social media platforms are defined as Web 2.0-based applications that enable the creation and exchange of user-generated content (Kim et al., 2015; Xu et al., 2021). In line with this, the current study demonstrates that platforms such as Tasteme and Homemakers’ Alliance in Taiwan play a crucial role in raising consumer awareness of sustainable food issues. These platforms enhance trust and user engagement by offering educational content and user-friendly interfaces.

However, when compared to European platforms such as Too Good To Go or OLIO, Taiwanese platforms still face challenges in real-time interaction and service completeness (Cappelli et al., 2022; Michelini et al., 2020; O'Neill et al., 2023; Simeone and Scarpato, 2020). These findings echo those of Wang (2021), who emphasized that user experience is fundamental to building trust in sustainable platforms. Enhancing interactivity and technical functionality will be key to maximizing the impact of such platforms in the future.

(2) Technological innovation and digital transformation

Although more than 85% of survey respondents acknowledged the value of sustainable food practices, a significant gap remains between knowledge and actual behavior (Hsu et al., 2020; Zamuz et al., 2021). This discrepancy aligns with Ajzen’s (1991) Theory of Planned Behavior, which posits that the misalignment between attitudes, subjective norms, and perceived behavioral control can prevent action.

This study highlights the importance of education in closing this gap. Embedding structured, gamified, or reward-based learning modules (e.g., carbon footprint calculators or eco-points systems) into platforms can deepen engagement and stimulate behavioral transformation (Mehrabi et al., 2022). These results are consistent with Lin (2018), who argued that “education-as-interface” models enhance user commitment in sustainability-related applications.

(3) Sustainable development and environmental protection

Trust is the foundation of digital platform transactions. Factor analysis results from this study show that information transparency (e.g., food certification reports, sourcing details) is a critical determinant of consumer trust, particularly among users with higher education levels (Gallo et al., 2023; Lewis, 2018; Ma and Chang, 2024).

These findings reinforce the trust-and-perceived-risk model proposed by Gefen (2003), which posits that visible product data enhances user trust in online settings. Furthermore, interviewees indicated frustration with the lack of real-time customer support and confirmation notifications on some platforms—challenges also observed in B2C e-commerce studies, such as those by Chen and Barnes (2007) (Zoli, 2021). This highlights the need for platforms to strengthen transparency and communication to build confidence in decision-making.

(4) Policy and regulatory environment

Although the one-way ANOVA indicated that occupation was significantly associated with differences in platform demand (*F*[10, 245] = 2.281, *p* = 0.014), the subsequent Tukey *post hoc* analysis revealed that this effect was not broadly distributed across all occupational groups. Instead, only the following two specific pairwise comparisons reached statistical significance at the *α* = 0.05 level:

- Military/Public Service/Education vs. Finance/Insurance (*p* = 0.018).
- Retired vs. Finance/Insurance (*p* = 0.017).

Most other occupational comparisons yielded non-significant *p*-values, with many exceeding 0.10 or even approaching 1.00, indicating that the occupational effect should be interpreted as localized rather than systemic. In this regard, the significant ANOVA result reflects the presence of isolated group differences rather than a generalized occupational stratification in platform demand (Liu et al., 2023; Michelini et al., 2018).

More specifically, respondents working in the finance and insurance sector consistently demonstrated higher levels of demand for sustainable food platforms compared to individuals from the public-sector/education/military groups and retired populations. This pattern may reflect differences in income stability, familiarity with digital services, and perceived financial flexibility across these groups. However, since most occupational categories did not differ significantly from one another, the influence of occupation should be understood as selective and context-dependent, rather than representing a broad structural determinant.

This refined interpretation aligns with prior findings suggesting that occupational effects on sustainable consumption are often mediated by income security, technological familiarity, and life-stage factors, rather than occupation per se (O'Neill et al., 2023).

(1) Globalization and market opportunities

Although food waste and food justice are globally recognized issues, platform design must reflect local cultural and environmental contexts. This study compared the structural logic of four platforms—Tasteme (Taiwan), Too Good To Go (Denmark), No Food Wasted (Netherlands), and OLIO (UK)—and found distinct interactive strategies aligned with each region’s socio-cultural values (Lawo et al., 2021; Mu et al., 2019).

**Figure 6:**
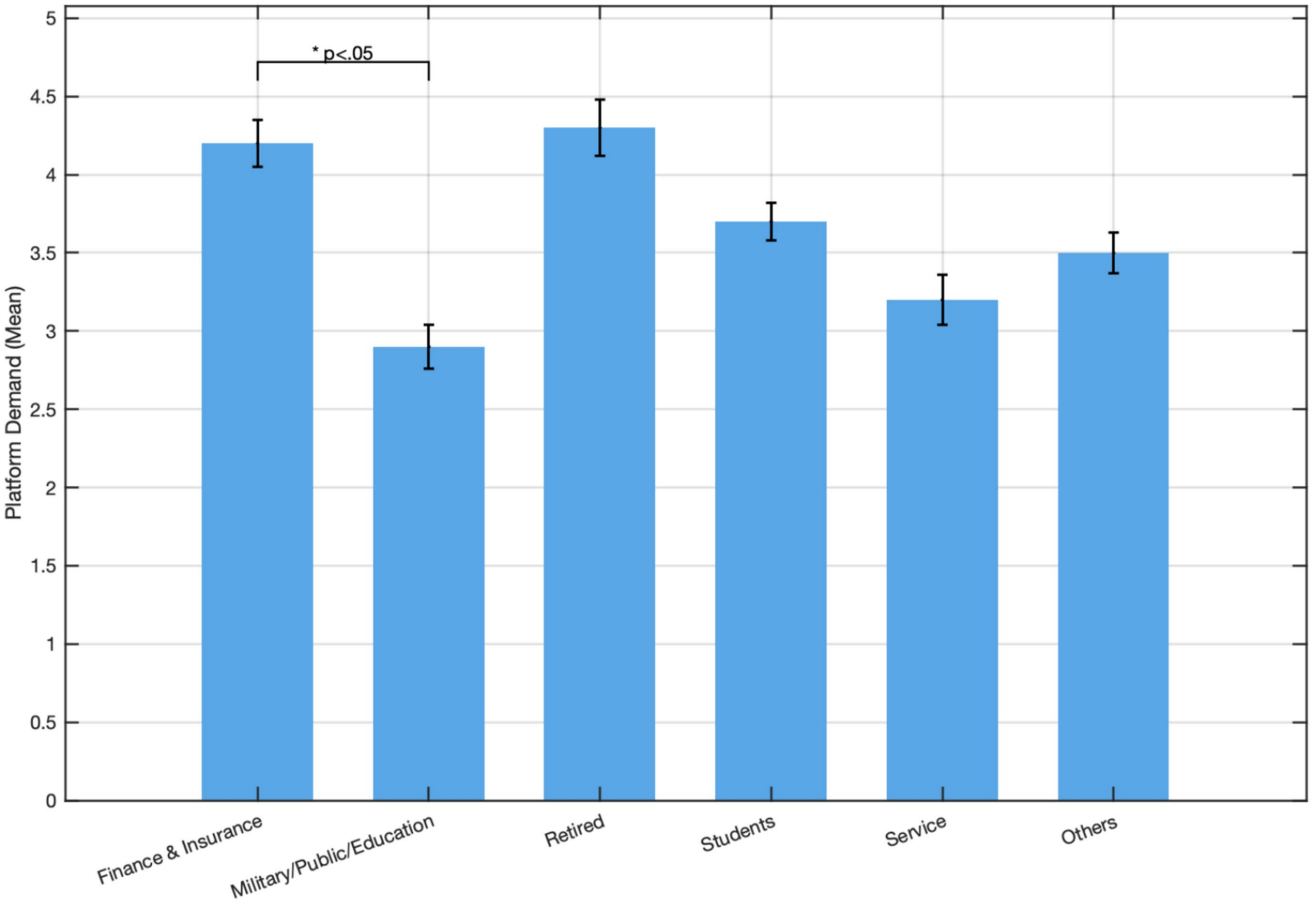
RGroup differences in platform demand (ANOVA).

For instance, Denmark focuses on technological innovation, the UK promotes neighborhood sharing, and Taiwan emphasizes “food appreciation” and eco-literacy. These findings reflect Hofstede’s (2001) cultural dimensions, especially the contrasts between collectivism and individualism or long-term vs. short-term orientation. Therefore, any cross-regional platform expansion must be grounded in cultural empathy and behavioral insight to optimize relevance and adoption, as shown in Figure 6 (Fuentes et al., 2021; Hyland et al., 2024; Kirmani et al., 2023).

These results align with the theoretical foundations of the Importance–Performance Analysis (IPA) method and echo the assumptions of the SERVQUAL service quality model and the Theory of Planned Behavior (TPB) regarding the relationship between perceived value and behavioral intention. Notably, Reliability Information is a critical driver within the trust dimension, underscoring that the transparency and credibility of information are vital for consumer decision-making on sustainable platforms.

Practical implications are as follows:

(1) Priority Improvement: Allocate resources to enhance the transparency of Reliability Information, such as implementing real-time verification systems, third-party endorsements, and comprehensive traceability mechanisms.
(2) Maintain Competitive Advantage: Continue improving the quality and coverage of Product Certificates and Educational Content to strengthen brand image and differentiation.
(3) Resource Reallocation: Assess the cost-effectiveness of investments in Innovative Ways and More Options to prevent over-concentration of resources in low-importance areas.

**Figure 7:**
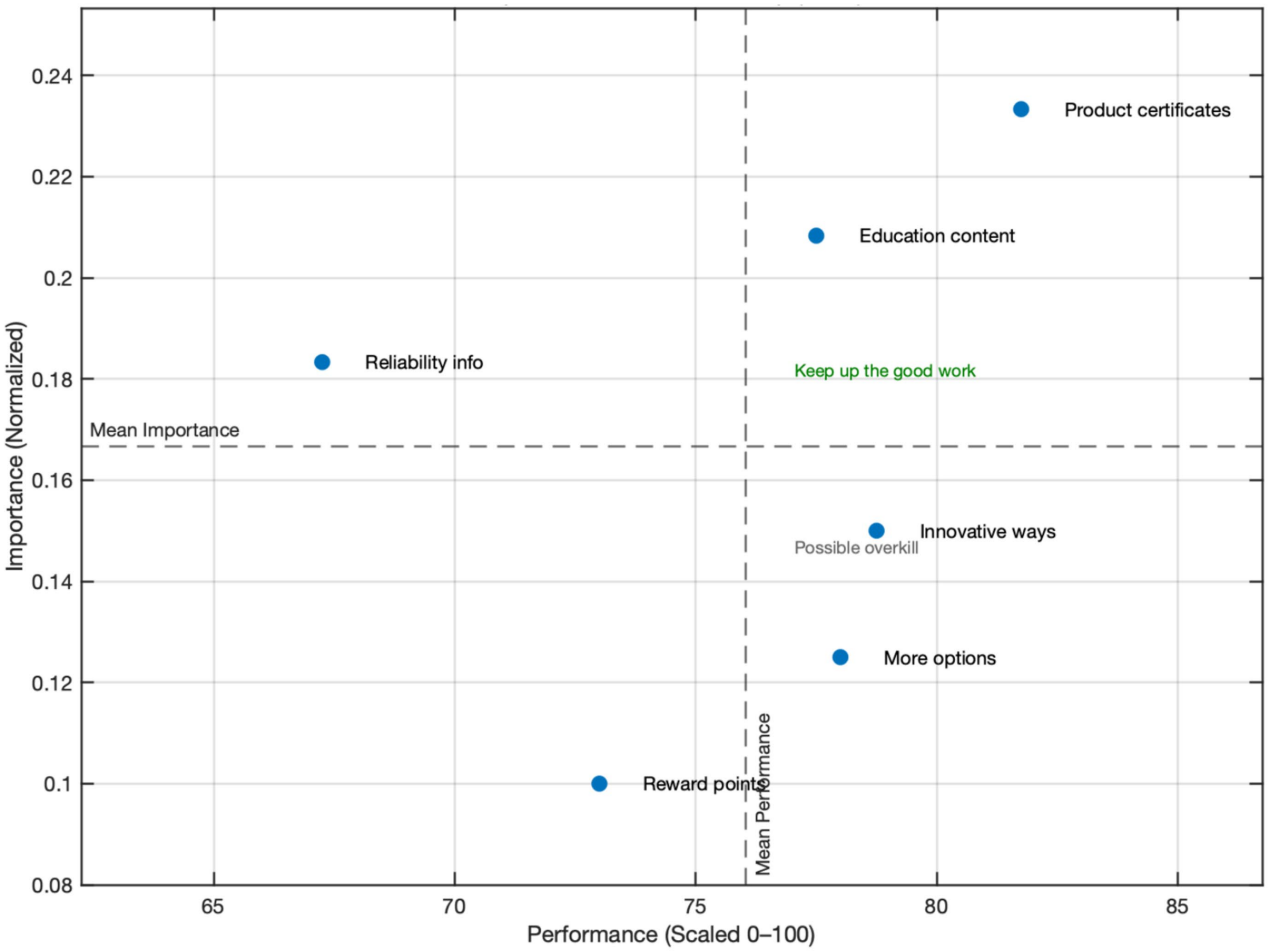
Importance–performance map analysis (IPMA).

The IPMA analysis shows that product certificates and educational content fall within the high-importance, high-performance quadrant, forming the core strengths of sustainable food platforms; reliability Information is of high importance but underperforms and should be prioritized for improvement; innovative ways and more options fall within the low-importance, high-performance quadrant, suggesting a moderate adjustment of resource allocation; and reward points resides in the low-importance, low-performance quadrant and is more suitable as a supplementary marketing tool. These findings not only provide clear guidance for resource allocation and functional optimization but also validate the critical role of information transparency and trust in shaping sustainable consumption behavior, as shown in Figures 6 and 7.

This study focuses on relational patterns (e.g., mediation and moderation effects, group differences, and conditional mechanisms) rather than on descriptive mean values alone. These inferential results rely on variance and covariation across respondents, which are less sensitive to upward shifts in overall scale means. Moreover, the presence of theoretically consistent barriers—such as modest willingness to pay a price premium and the conditional effectiveness of rewards under high price salience—suggests that responses were not uniformly inflated by pro-sustainability attitudes. Finally, this study outlines future research directions, including probability-based sampling and experimental designs, to further mitigate self-selection concerns and strengthen causal inference.

This study demonstrates that consumer engagement with anti-food-waste platforms is primarily driven by trust in the platform trust and information transparency, rather than by reward incentives alone. Although eco-point systems were positively evaluated, their impact on behavioral intention proved strongly conditional. Specifically, under high perceived price salience, reward effectiveness significantly diminished, while trust-related cues (e.g., certification and traceability) remained stable predictors of platform loyalty.

These findings extend previous TPB-based research on sustainable consumption by illustrating that cost salience disrupts the motivation–intention link, highlighting a psychological tension between moral incentives and economic rationality. Importantly, the moderated mediation results reveal that highly educated users are less influenced by gamification and more dependent on informational credibility, confirming that cognitive evaluation overrides emotional rewards in high-literacy groups.

Furthermore, occupational differences, although statistically significant, showed small-to-moderate effect sizes and limited pairwise significance. This suggests that psychological variables outweigh structural demographics in shaping platform demand. Consequently, design strategies should prioritize strengthening trust architecture—such as real-time verification systems and certification displays—over expanding superficial gamification mechanisms.

### 4.1 Limitations and recommendations

This study explores the impact of sustainable food platforms on consumer awareness from the perspective of consumer cognition. It analyses and discusses existing sustainable food platforms to address the issue of surplus food in Taiwan, China. The research scope and limitations of this study are outlined in the following:

(1) This study focused on sustainable food. Other food-related issues, such as food security and the impact of climate on food, were not involved to maintain a clear research focus.
(2) The geographical scope of this study was limited to Taiwan, considering regional differences in culture, food consumption habits, and social media usage. This helps ensure the practicality of these research findings in the sustainable food scenarios of Taiwan.
(3) Results from the moderated mediation model revealed that reward sensitivity significantly influences platform trust formation, which in turn enhances platform loyalty and continuous usage intention. However, this effect is significantly weakened under high price salience and among highly educated users, indicating that eco-point rewards function primarily as supportive rather than primary motivators when economic or cognitive evaluation pressures are strong.

Although prior studies on sustainable food consumption and platform-mediated food redistribution have adopted diverse theoretical perspectives—such as the Theory of Planned Behavior (TPB), technology trust models, Hofstede’s cultural dimensions, SERVQUAL, and gamification theory—these frameworks have often been applied in isolation. This fragmentation has led to scattered interpretations of consumer behavior in anti-food-waste platforms, making it difficult to construct a coherent psychological explanation of platform adoption and continuance.

To address this limitation, the present study develops an integrated psychological decision framework by combining four theoretically complementary streams:(1) the Theory of Planned Behavior (TPB); (2) platform trust theory; (3) gamification and motivational design, and (4) service quality and value perception perspectives.

## 5 Conclusion

The present study found that cost salience was perceived as a relatively strong barrier to the adoption of sustainable food platforms (*M* = 3.32), whereas platform reliability information, such as certification transparency and traceability disclosure, received the highest overall evaluation (*M* = 4.27). However, it is important to clarify that conclusions regarding the functional role of eco-points and reward incentives cannot be drawn from descriptive statistics and importance–performance map analysis (IPMA) alone.

When incorporating the moderation analysis results, the findings provide more nuanced evidence for the motivational role of rewards. Specifically, although eco-points were not positioned as a primary driver of adoption, their positive influence on platform usage intention was conditional rather than universal. Reward sensitivity exerted a significant effect on behavioral intention only under conditions of low-cost salience, and this effect weakened or disappeared among users with high perceived price pressure and higher educational attainment.

Therefore, eco-points should be more accurately described as supportive and conditional motivators that enhance platform engagement only when economic concerns are not dominant and when trust has been sufficiently established. In contrast, information reliability and transparency serve as more stable, foundational drivers across user groups. This refinement strengthens the study’s central argument that “rewards align—only under specific psychological and contextual conditions.”

In summary, the effective design and promotion of sustainable food platforms can not only strengthen consumers’ awareness of sustainable food but also motivate them to choose sustainable food products. This further promotes the development of a green economy and sustainable lifestyles, and further drives the operation and growth of the sustainable industry. By presenting clear information, such as food reports and surplus food sources, and combining key factors, such as education, reward systems, and convenience, platforms can change consumers’ purchasing behavior and make them more inclined to choose environmentally friendly and healthy products. The discussion of sustainable food platforms in this study is expected to provide key references and directions for consumer behaviors and for formulating brand marketing strategies.

## Data Availability

The datasets presented in this study can be found in online repositories. The names of the repository/repositories and accession number(s) can be found in the article/Supplementary material.
